# Cognitive changes associated with switching to frequent nocturnal hemodialysis or renal transplantation

**DOI:** 10.1186/s12882-016-0223-9

**Published:** 2016-01-22

**Authors:** Bradley S. Dixon, John M. VanBuren, James R. Rodrigue, Robert S. Lockridge, Robert Lindsay, Christopher Chan, Michael V. Rocco, Jacob J. Oleson, Leigh Beglinger, Kevin Duff, Jane S. Paulsen, John B. Stokes, the FHN study

**Affiliations:** Department of Internal Medicine, Carver College of Medicine, University of Iowa, Iowa City, IA USA; Veterans Administration Medical Center, Iowa City, IA USA; Department of Biostatistics, College of Public Health, University of Iowa, Iowa City, IA USA; Center for Transplant Outcomes and Quality Improvement, The Transplant Institute, Beth Israel Deaconess Medical Center and the Harvard Medical School, Boston, MA USA; Department of Internal Medicine, University of Virginia Health System, Charlottesville, VA USA; Department of Medicine, The University of Western Ontario, London, ON Canada; Department of Medicine, University of Toronto, University Health Network, Toronto, ON Canada; Department of Medicine, Wake Forest School of Medicine , Winston-Salem, NC USA; Departments of Psychiatry, Neurology and Psychology, Carver College of Medicine, University of Iowa, Iowa City, IA USA; Departments of Psychology and Neurology, University of Utah, Salt Lake City, UT USA; Nephrology Division, University of Iowa School of Medicine, E300D GH, 200 Hawkins Drive, Iowa City, IA 52242-1081 USA

**Keywords:** Renal replacement therapy, Frequent nocturnal hemodialysis, Renal transplantation, Cognitive impairment, Cognition, Neuropsychological testing

## Abstract

**Background:**

It is uncertain whether switching to frequent nocturnal hemodialysis improves cognitive function in well-dialyzed patients and how this compares to patients who receive a kidney transplant.

**Methods:**

We conducted a multicenter observational study with longitudinal follow-up of the effect on cognitive performance of switching dialysis treatment modality from conventional thrice-weekly hemodialysis to frequent nocturnal hemodialysis, a functioning renal transplant or remaining on thrice-weekly conventional hemodialysis. Neuropsychological tests of memory, attention, psychomotor processing speed, executive function and fluency as well as measures of solute clearance were performed at baseline and again after switching modality. The change in cognitive performance measured by neuropsychological tests assessing multiple cognitive domains at baseline, 4 and 12 months after switching dialysis modality were analyzed using a linear mixed model.

**Results:**

Seventy-seven patients were enrolled; 21 of these 77 patients were recruited from the randomized Frequent Hemodialysis Network (FHN) Nocturnal Trial. Of these, 18 patients started frequent nocturnal hemodialysis, 28 patients received a kidney transplant and 31 patients remained on conventional thrice-weekly hemodialysis. Forty-eight patients (62 %) returned for the 12-month follow-up. Despite a significant improvement in solute clearance, 12 months treatment with frequent nocturnal hemodialysis was not associated with substantial improvement in cognitive performance. By contrast, renal transplantation, which led to near normalization of solute clearance was associated with clinically relevant and significant improvements in verbal learning and memory with a trend towards improvements in psychomotor processing speed. Cognitive performance in patients on conventional hemodialysis remained stable with the exception of an improvement in psychomotor processing speed and a decline in verbal fluency.

**Conclusions:**

In patients on conventional thrice-weekly hemodialysis, receiving a functioning renal transplant was associated with improvement in auditory-verbal memory and psychomotor processing speed, which was not observed after 12 months of frequent nocturnal hemodialysis.

**Electronic supplementary material:**

The online version of this article (doi:10.1186/s12882-016-0223-9) contains supplementary material, which is available to authorized users.

## Background

Cognitive function is impaired in people with chronic kidney disease (CKD) and correlates with the degree of kidney dysfunction [1]. In patients with CKD, lower cognitive function is associated with higher rates of mortality, utilization of health care resources and a lower quality of life [2–4]. The etiology of cognitive dysfunction in patients with CKD is multifactorial including the uremic environment, fluid shifts with dialysis, anemia, medications, depression, acute illness and structural lesions in the brain associated with progressive vascular disease [4–6]. Previous studies have shown that renal replacement therapy with dialysis or transplantation is associated with improvement in cognitive function [7–9]. Patients who receive a renal transplant have been reported to achieve cognitive function close to normal controls [10]. However, patients on conventional dialysis, even in the era of high-flux dialysis, have been reported to have persistent cognitive deficits [11–14]. Recent studies have suggested that much of this cognitive dysfunction is associated with vascular disease and may not be reversible with further increases in solute clearance [13, 15, 16].

Compared to conventional thrice-weekly hemodialysis, frequent hemodialysis 6 days a week increases urea clearance, produces less fluctuation in fluid balance and improves control of blood pressure and serum phosphate, which might further improve cognitive function. A small, uncontrolled study found that conversion from conventional thrice-weekly hemodialysis to frequent nocturnal hemodialysis was associated with improvement in cognitive performance at 6 months [17]. Improvements were seen in psychomotor efficiency and processing speed as well as in attention and working memory. However, these patients were significantly younger and healthier than the typical hemodialysis patient. The effect of frequent hemodialysis on cognition was further studied in the Frequent Hemodialysis Network (FHN) Trials, which consisted of two trials: the Daily Trial that randomized patients with end stage renal disease (ESRD) to treatment with conventional in-center thrice-weekly hemodialysis versus in-center hemodialysis 6 days a week and the Nocturnal Trial which randomized patients to home or in-center thrice-weekly hemodialysis versus nocturnal hemodialysis 6 times per week [18]. Despite increased intensity of dialysis, the FHN Trials found no improvement in executive function or global cognition in either Trial [19]. However, exploratory analyses in a subset of patients who underwent more detailed cognitive testing revealed improvements in memory and verbal fluency in the Daily Trial that were not observed in the Nocturnal Trial [19].

In contrast to frequent hemodialysis, renal transplantation restores renal function to near normal and provides a better measure of the reversibility of cognitive deficits seen in patients on conventional hemodialysis. We therefore extended the cognitive ancillary study of the FHN Nocturnal Trial to include patients who received a functioning kidney transplant and added additional patients outside of the FHN Trial who initiated nocturnal hemodialysis or remained on conventional thrice-weekly hemodialysis. The present observational study explored changes in cognitive function at 4 and 12 months compared to baseline after switching to nocturnal hemodialysis 6 days a week, receiving a functioning renal transplant or remaining on conventional thrice-weekly hemodialysis.

## Methods

### Patient population

Patients on conventional thrice-weekly hemodialysis at one of the participating centers who were eligible to switch to frequent nocturnal home hemodialysis or were on the kidney transplant list were invited to participate. The initial 21 of 77 patients were recruited from the Frequent Hemodialysis Network (FHN) Nocturnal Trial at one of the five study sites in North America [20]. Details of the FHN Trials have been published [18]. Some details on the cognitive effects of frequent nocturnal hemodialysis for these 21 patients (9 control and 12 nocturnal) randomized in the Nocturnal Trial have previously been published [19]. One patient randomized to the nocturnal group, returned to conventional thrice-weekly hemodialysis before the 4-month testing and was included “as-treated” in the conventional dialysis group in the present study. After the FHN Trial closed, patients on conventional hemodialysis at a participating site who started frequent nocturnal hemodialysis and met entry criteria for the FNH Trial continued to be recruited to participate in the non-randomized cognitive study. Subjects for the transplant arm were recruited from hemodialysis patients at the top of the transplant waiting list and expected to receive either a deceased donor or living donor kidney within the next month. Patients for the conventional arm of the cognitive study were recruited from hemodialysis patients on the renal transplant waiting list and not expected to receive a transplant within the next year. The patients recruited for the conventional hemodialysis study arm were matched as closely as possible for baseline characteristics including age, gender and race of those in the other two study arms. The trial was approved by the local Institutional Review Board (IRB) at each participating site (see Additional file 1). All participants signed an IRB-approved consent to participate in this observational study. The study adhered to the Declaration of Helsinki. The FHN Nocturnal trial was registered at Clinical Trials.gov #NCT 00271999 on 1/6/2006.

### Study factor

Participants who met the inclusion and exclusion criteria for the FHN Trial [18] were randomized to home nocturnal hemodialysis for ≥6 h per night, 6 nights per week to achieve a weekly target stdKt/V ≥ 4.0/week or remained on conventional thrice-weekly hemodialysis ≥2.5 h per session with a prescribed eKt/V > 1.1 and stdKt/V ≥ 2.0 [20]. After the FHN Trial closed, additional patients who started frequent nocturnal hemodialysis or remained on conventional hemodialysis were enrolled using the same inclusion and exclusion criteria into the extended cognitive observational study and underwent the same intervention and follow-up as for the FHN Trial. Participants in the transplant group underwent kidney transplant per the standard of care in their transplant program.

### Study design

Baseline data collection and cognitive testing were performed within the month prior to the planned date of the starting frequent nocturnal hemodialysis or getting a renal transplant. A second baseline study was performed if there was a delay in switching modality. Follow-up cognitive testing was scheduled for 4 and 12 months after starting frequent nocturnal hemodialysis or getting a renal transplant or 4 and 12 months after the baseline study in the group that remained on conventional thrice-weekly hemodialysis. Cognitive testing was done in the morning before noon on a non-dialysis day for all patients on conventional hemodialysis. Cognitive testing for patients on nocturnal hemodialysis was done in the morning at least 2 h after completing hemodialysis. Cognitive testing for patients who received a transplant could be on any morning before noon.

### Neuropsychological testing

Cognitive testing was conducted in a quiet room, free of distractions using a standardized set of procedures and scripted language by an examiner trained and supervised by a neuropsychologist who was very familiar with each test and the study protocol. The testing required about 90 min and consisted of both computer tasks as well as pencil and paper tests. A description of the neuropsychological studies performed and cognitive domains tested is shown in Table 1. Where available, alternate test forms designed to limit practice effects were used for each follow-up visit.

**Table 1:** Neuropsychological tests performed

Test name	Cognitive domain(s)	Brief Description
American National Adult Reading Test (ANART)	Estimated premorbid Intelligence	Ability of subject to properly pronounce a list of 50 irregular words.
Modified Mini-Mental Status Exam (3MS)	Dementia and global cognitive function	Series of questions testing orientation, memory, arithmetic ability, attention, repetition, language and visuospatial drawing ability.
Beck Depression Inventory (BDI-II)	Depression	Multiple choice 21 item questionnaire assessing severity of depression
Auditory Verbal Learning Test (AVLT)	Verbal learning and memory	Tests subject’s ability to correctly recall a set of 15 words presented verbally 5 times to test auditory learning. After a distractor set of 15 different words the subject is asked to recall the original 15 words either immediately (immediate recall) or after a delay of 30 min (delayed recall).
Brief Visuospatial Memory Test –Revised (BVMT-R)	Visuospatial learning and memory	Tests subject’s ability to learn and remember 6 geometric figures and their corresponding spatial locations from a printed display. The display is presented three times with free recall (drawing) after each presentation. After approximately 25 min, the participant is again asked to draw the display.
Letter-Number Sequencing	Working memory and attention	Subject is verbally presented a list of numbers (N) and letters (L) that they must order (and verbally recite) correctly. Starts with 3 different sets of 2 N + L combos and increases to 3 sets of 8 N + L combos.
N-Back (Computer task)	Working and short-term memory	Subject is presented with a sequence of stimuli, and the task consists of indicating when the current stimulus matches the one from 2 steps earlier in the sequence. Lures (rather than foils) are used in some tests where the stimulus presented matches one from either 1 or 3 steps (but not 2 steps) back.
Digit Symbol Test	Psychomotor processing speed, working memory	Tests subject’s ability to correctly match and write down a number corresponding with a particular symbol. Score is number of items completed correctly in 90 s.
Chooser (Choice Reaction Time) (Computer task)	Psychomotor processing speed and attention	Tests subject’s reaction time moving finger from home button to one of two alternative signal buttons that has been lit up.
Buttons (Motor Tracking) (Computer task)	Ability to utilize advanced information (planning) to improve psychomotor processing speed and attention	Tests subject’s reaction time in repeatedly releasing their finger from the home button and correctly moving to one of two alternative buttons in separate columns that become lit up. Varying levels of cues are offered to determine whether subject is able to use additional information to improve performances.
Trail Making Test forms A and B	Psychomotor processing speed and executive function (problem solving, planning, organizational skills, selective attention, inhibitory control, working memory)	Trails A measures time required to connect numbers scattered on a page in correct sequential order. Trails B measures time required to connect a series of numbers (1–12) and letters (A-L) in correct consecutive sequential order (1-A-2-B, etc.).
Controlled Oral Word Association Test (COWAT)	Verbal fluency, flexibility and initiation. Language	Subject is given a certain letter (such as “F”) and must say as many words as they can think of that start with that letter in 60 s. This is repeated 2 more times with different letters.

### Analytical approach

Standard descriptive statistics were used to compare baseline demographic and clinical characteristics between the three groups. A longitudinal linear regression model with an unstructured covariance matrix allowing for correlation between multiple visits was used to evaluate the change in each cognitive test over time. Preliminary analyses demonstrated that some cognitive tests exhibited both a significant within group change over time and a significant difference between groups on follow-up so a ‘group by follow-up time’ interaction term was included in the model. In addition to intervention group, follow-up time, and the ‘group by follow-up time’ interaction, selected baseline demographic and clinical variables were included in the adjusted model. Baseline variables that associated with each cognitive test at a univariate significance of 0.10 or less were subsequently included in a multiple regression analysis and Bayesian Information Criteria was used to select the final variables included in the adjusted model. The baseline variables selected for the regression model on each cognitive test are listed in Additional file 2: Table S1. Log transformation was performed for Trails A and Trails B tests and the Chooser task to approximate normality. All statistical analyses were conducted using SAS, v9.3. All *p*-values are two-sided and were not corrected for multiple testing. A *p*-value ≤ 0.05 was considered statistically significant.

## Results

### Participant characteristics

Seventy-seven patients were enrolled, 21 patients (9 control and 12 nocturnal) were recruited from the randomized FHN Trial and the remaining 56 patients were enrolled outside of that Trial. A total of 18 patients underwent frequent nocturnal hemodialysis, 28 patients underwent kidney transplantation and 31 patients remained on conventional thrice-weekly hemodialysis. The baseline demographic and clinical characteristics are shown in Table 2. For the combined group the average age was 49 years old, 38 % were female, 58 % had at least some college education, and 5 % had suffered a stroke. The mean (±SD) Beck Depression Inventory score was 11.9 ± 8.3 indicative of mild depression and the premorbid group IQ estimated by the ANART score of 25.1 ± 12.3 was slightly above the population average. At baseline, the average single pool Kt/V was 1.49 ± 0.41, the serum albumin was 4.0 ± 0.54 mg/dl and the hemoglobin was 11.7 ± 1.5 g/dL. Histamine H1 antagonist use was reported in 11.7 %, while 9.1 % reported using a sedative-hypnotic medication and 24.7 % were prescribed a narcotic. There was no statistically significant difference between the three intervention groups in any baseline parameter except the use of vitamin D analogs.

**Table 2:** Baseline Demographics

Characteristic^a^	Control	Nocturnal	Transplant	*p*
Total Participants (*N*)	31	18	28	
Gender (female, %)	32	39	43	0.698
Age (years)	49.5 ± 15.4	47.9 ± 14.7	49.93 ± 12.75	0.894
Education				0.099
High School Diploma or Less (%)	48	50	29	
Some College (%)	26	44	32	
Bachelor’s Degree or More (%)	26	6	39	
Race				0.303
Caucasian (%)	68	50	71	
Other (%)	32	50	29	
Household Income				0.442
< $20 k	39	28	32	
$20 k - $49 k	22	50	32	
>$50 k	29	11	18	
Unknown/Refused	10	11	18	
Diabetes (%)	35	39	25	0.555
Stroke (%)	3.2	11	3.6	0.433
Congestive Heart Failure (%)	9.7	16.7	3.6	0.317
Smoking				0.464
Never	55	61	57	
Used To	23	28	36	
Currently	23	11	7	
Beck Depression Inventory-2	14.4 ± 10.6	10.1 ± 5.1	10.3 ± 6.6	0.123
ANART Raw Score	26.3 ± 12.7	26.7 ± 12.0	22.7 ± 12.1	0.457
Modified Mini-Mental Status	85.2 ± 7.6	88.1 ± 5.7	82.9 ± 15.6	0.293
Weekly Std Kt/V	1.38 ± 0.50	1.32 ± 0.32	1.37 ± 0.26	0.854
Pre Dialysis BUN (mg/dL)	55 ± 15	47 ± 15	61 ± 25	0.064
Pre Dialysis Creatinine (mg/dL)	9.0 ± 3.7	8.0 ± 3.0	9.4 ± 3.8	0.407
Pre Dialysis Albumin (g/dL)	4.1 ± 0.5	3.8 ± 0.7	4.1 ± 0.5	0.154
Pre Dialysis Weight (Kg)	81.0 ± 20.2	97.1 ± 29.6	88.9 ± 23.9	0.082
Pre Dialysis Phosphorus (mg/dL)	5.4 ± 1.6	5.3 ± 1.8	5.1 ± 1.6	0.707
Hemoglobin (g/dL)	11.5 ± 1.3	11.5 ± 1.3	12.0 ± 1.8	0.375
Pre Dialysis Systolic Blood Pressure (mmHg)	147 ± 27	143 ± 31	142 ± 25	0.791
Pre Dialysis Diastolic Blood Pressure (mmHg)	80 ± 16	79 ± 19	81 ± 16	0.899
Ultra Filtration Volume (L)	2.3 ± 1.7	2.4 ± 1.5	2.9 ± 1.5	0.355
Urea Reduction Ratio (%)	71 ± 9	70 ± 8	72 ± 6	0.606
Vitamin D use (%)	23	63	32	0.027
Medication				
H1 Receptor antagonist (%)	10	17	11	0.749
Narcotic (%)	19	28	29	0.672
Muscle Relaxant (%)	6.5	5.6	0	0.405
Anti-Convulsant (%)	29	17	11	0.195
Anti-Depressant (%)	23	28	29	0.855
Anti-Emetic (%)	10	17	11	0.749
Anti-Psychotic (%)	3.2	0	7.1	0.460
Anxiolytic (%)	10	11	25	0.225

^a^Continuous variables expressed as mean ± SD

**Fig. 1:**
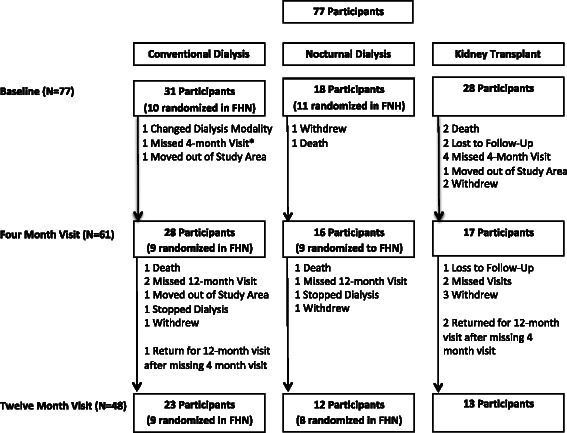
Patient flow diagram

Forty-eight patients (62 %) returned for the 12-month follow-up, 12 patients (67 %) in the nocturnal hemodialysis group, 13 patients (46 %) in the transplant group and 23 patients (74 %) on conventional hemodialysis. Patient flow and reasons for dropout are shown in Fig. 1. The characteristics of the three groups at 12 months are shown in Table 3. As expected, the weekly stdKt/V was significantly higher in the group on frequent nocturnal hemodialysis (5.56 ± 1.12) compared to those on conventional hemodialysis (2.27 ± 0.21, *p* < 0.001). At 12 months, the serum creatinine and phosphorus levels were significantly lower in patients on frequent nocturnal dialysis compared to conventional hemodialysis and were reduced significantly further in patients who received a kidney transplant. Patients who received a kidney transplant also had significantly higher hemoglobin levels. Otherwise there were no statistically significant differences (including sedative medications) between the three groups.

**Table 3:** 12-month follow-up

Characteristic^a^	Control	Nocturnal	Transplant	*p*
Total Participants (*N*)	23	12	13	
Gender (female, %)	26	25	46	0.397
Age (years)	47.2 ± 13.6	49.9 ± 14.9	48.8 ± 12.7	0.843
Caucasian (%)	70	42	77	0.167
Diabetes (%)	22	30	15	0.802
Stroke (%)	0	10	0	0.222
Congestive Heart Failure (%)	4	10	0	0.466
Currently smoking (%)	26	0	15	0.429
Beck Depression Index	11.9 ± 10.2	8.8 ± 5.9	7.7 ± 6.2	0.322
Modified Mini-Mental Status	89.4 ± 4.9	90.3 ± 4.7	89.8 ± 4.3	0.877
Weekly Std Kt/V	2.27 ± 0.21	5.56 ± 1.15	NA	0.001*
Pre Dialysis BUN (mg/dL)	61 ± 17	43 ± 14	NA	0.008*
Pre Dialysis Creatinine (mg/dL)	10.1 ± 2.8	7.1 ± 3.1	1.5 ± 0.6	0.001*
Pre Dialysis Albumin (g/dL)	4.1 ± 0.5	4.0 ± 0.4	4.5 ± 0.4	0.136
Pre Dialysis Phosphorus (mg/dL)	5.8 ± 1.6	4.3 ± 1.6	2.7 ± 0.4	0.001*
Hemoglobin (g/dL)	11.9 ± 1.5	11.3 ± 2.1	13.8 ± 1.2	0.001*
Pre Dialysis Systolic Blood Pressure (mmHg)	149 ± 26	135 ± 17	NA	0.123
Pre Dialysis Diastolic Blood Pressure (mmHg)	84 ± 12	76 ± 12	NA	0.098
Ultra Filtration Volume (L)	2.9 ± 1.4	2.2 ± 1.0	NA	0.179
Urea Reduction Ratio (%)	73 ± 8	78 ± 14	NA	0.158
Vitamin D use (%)	27	17	23	0.905
Medication				
H1 Receptors (%)	13	8	8	1.000
Narcotic (%)	39	25	23	0.561
Muscle Relaxant (%)	0	8	0	0.250
Anti-Depressant (%)	39	33	31	0.926
Sedative-hypnotic (%)	17	0	0	0.170
Anxiolytic (%)	13	8	8	1.000
Patient Hospitalized (%)	30	42	15	0.357

^a^Continuous variables expressed as mean ± SD*P<0.05

### Effect of intervention on cognition

**Fig. 2:**
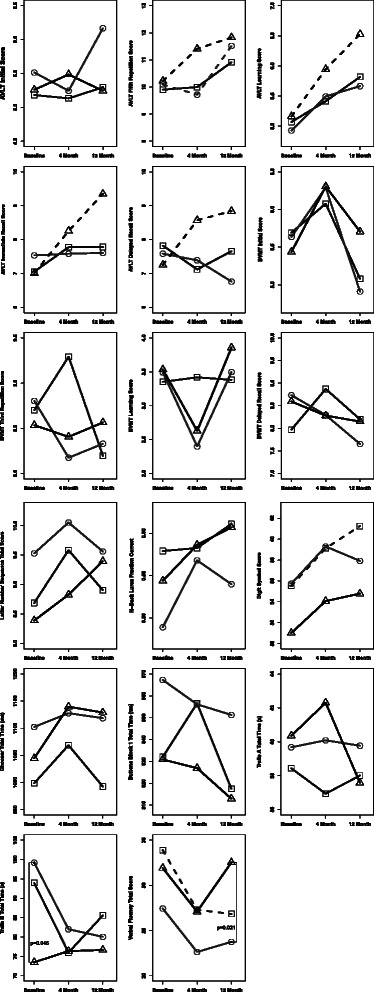
Change in selected cognitive tests at 4 and 12 months after switching to frequent nocturnal hemodialysis (*circles*, ○) receiving a renal transplant (*triangles*, ∆) or remaining on conventional thrice-weekly hemodialysis (*squares*, □). Data shows analysis after adjustment for difference in baseline characteristics as described in Additional file 2: Table S1. Refer to Table 1 and legend to Table 4 for a description of the tests and abbreviations. Dashed lines represent statistically significant (*p* < 0.05) within group changes in test performance between 12 months and baseline. Vertical lines with the associated *p*-value represent statistically significant (*p* < 0.05) pairwise differences between study groups at the specified study visit

As shown in Table 4 and Fig. 2, treatment with frequent nocturnal hemodialysis for 12 months was associated with a borderline significant, modest improvement in the number of words recalled on trial 5 of the Auditory Verbal Learning Test (AVLT-T5, percent change between 12 months and baseline = 14.8 %, *p* = 0.048) and a significant but modest decline in delayed recall on the Brief Visuospatial Memory Test (BVMT-DR, percent change = −18.5 %, *p* = 0.018). No statistically significant differences occurred over 12 months in any of the other cognitive tests when compared to baseline.

**Table 4:** Effect of study intervention on cognitive testing after 12 months compared to baseline

Test^a^	Cognitive Domain	Study Group	Percent Change (12 mon – Baseline)/(Group Baseline Average)^b^	*P*-value	Group x Time Interaction
					*P*-value
AVLT_LA_T1	Memory (auditory)	Control	1.8	0.726	
		Nocturnal	13.8	0.307	0.743
		Transplant	−1.5	0.965	
AVLT_LA_T5	Memory (auditory) & Learning	Control	9.5	0.081	
		Nocturnal	14.8	0.048*	0.332
		Transplant	20.9	0.003*	
AVLT Learning Score	Memory (auditory) & Learning	Control	14.9	0.213	
		Nocturnal	9.9	0.681	0.609
		Transplant	35.9	0.009*	
AVLT_LA_IR	Immediate Recall Memory (auditory)	Control	16.2	0.151	
		Nocturnal	3.2	0.864	0.264
		Transplant	31.9	0.002*	
AVLT_LA_DR	Delayed Recall Memory (auditory)	Control	−0.6	0.825	
		Nocturnal	−5.4	0.242	0.021*
		Transplant	23.3	0.012*	
BVMT_T1	Memory (visual-motor)	Control	−10.0	0.191	
		Nocturnal	−11.7	0.203	0.619
		Transplant	5.6	0.565	
BVMT_T3	Memory (visual-motor) & Learning	Control	−4.7	0.341	
		Nocturnal	−9.2	0.221	0.215
		Transplant	0.9	0.933	
BVMT Learning Score	Memory (visual-motor) & Learning	Control	8.8	0.76	
		Nocturnal	−2.3	0.82	0.302
		Transplant	−2.2	0.558	
BVMT_DR	Delayed Recall Memory (visual-motor)	Control	6.1	0.439	
		Nocturnal	−18.5	0.043*	0.164
		Transplant	−3.6	0.500	
Letter Number Sequence	Working Memory	Control	5.2	0.594	
		Nocturnal	−0.9	0.982	0.277
		Transplant	10.2	0.082	
N-Back	Working Memory, Attention (Executive function)	Control	−2.3	0.093	
		Nocturnal	14.0	0.544	0.854
		Transplant	18.0	0.995	
Digit Symbol	Psychomotor processing speed, (Executive Function)	Control	13.4	0.000*	
		Nocturnal	3.7	0.305	0.682
		Transplant	12.2	0.054*	
Chooser (Choice Reaction Time)	Psychomotor processing speed	Control	2.0	0.070	
		Nocturnal	−0.6	0.752	0.591
		Transplant	10.1	0.586	
Buttons (Motor Tracking)	Psychomotor processing speed	Control	−0.0	0.369	
		Nocturnal	−1.2	0.946	0.558
		Transplant	−7.4	0.275	
Trails A	Psychomotor Processing Speed & attention	Control	4.8	0.649	
		Nocturnal	−0.2	0.907	0.660
		Transplant	−5.1	0.934	
Trails B	Executive Function	Control	−4.5	0.867	
		Nocturnal	−20.9	0.187	0.156
		Transplant	−3.0	0.311	
Verbal Fluency (COWAT)	Language & Executive Function	Control	−23.8	0.001*	
		Nocturnal	−14.6	0.080	0.017*
		Transplant	3.7	0.929	

^a^See Table 1 for details on cognitive tests. * P<0.05. AVLT_LA_T1, Auditory Verbal Learning Test, word list A, first administration; AVLT_LA_T5, Auditory Verbal Learning Test, word list A, fifth administration; AVLT Learning Score, represents the difference in the maximum number of words recalled on the 4^th^ or 5^th^ administration minus the baseline recall; AVLT_LA_IR, Auditory Verbal Learning Test, word list A, immediate recall; represents immediate recall from word list A after completion of the distractor list of 15 different words; AVLT_LA_DR, Auditory Verbal Learning Test, word list A, delayed recall; represents delayed recall from word list A, 30 min after completion of the distractor list of 15 different words; BVMT_T1, Brief Visuospatial Memory Test, first administration; BVMT_T3, Brief Visuospatial Memory Test, third administration; BVMT Learning Score, measures the mean difference in the maximum and baseline performance on the BVMT at that study visit; BVMT_DR, Brief Visuospatial Memory Test, delayed recall, measures recall of the original figures following a 25-min delay

^b^Percent change was computed as the difference between the twelve month visit and baseline visit divided by the average baseline score for the intervention group

By contrast, treatment with kidney transplantation was associated with a statistically significant improvement in the Auditory Verbal Learning Test (AVLT-T5, percent change = 20.9 %, *p* = 0.003), the AVLT learning score (percent change = 35.9 %, *p* = 0.009), the AVLT immediate recall (AVLT-IR, percent change = 31.9 %, *p* = 0.002), and the AVLT delayed recall (AVLT-DR, percent change = 23.3 %, *p* = 0.012), as well as a trend towards significance in the Digit Symbol test (percent change = 12.2 %, *p* = 0.054). At 12 months, transplant patients also showed a significantly higher verbal fluency (COWAT) score compared with patients on nocturnal hemodialysis (*p* = 0.017).

The dropout rate was higher in the transplant group than the other two groups. In order to rule out dropout bias, an exploratory analysis was performed to compare baseline demographics within the transplant group between those who returned for at least one follow-up and those transplant participants who dropped out after baseline. The ANART score and serum albumin levels were significantly lower in the group who dropped out and considered a potentially clinically significant confounder. However, adjustment for these two variables in the model did not impact our results. In addition, an analysis limited to only those patients who completed 3 study visits led to the same conclusions.

Twelve months treatment with conventional hemodialysis was associated with a statistically significant improvement in the Digit Symbol test (percent change = 13.4 %, *p* < 0.001) and a statistically significant decline in verbal fluency (Controlled Oral Word Association Test, COWAT; percent change = −23.8 %, *p* < 0.001) compared to baseline. Otherwise performance on cognitive testing in patients on conventional hemodialysis remained stable over 12 months.

## Discussion

Despite significant improvement in the clearance of urea, creatinine and phosphorus, 12 months of treatment with frequent nocturnal hemodialysis did not lead to improvement in cognitive performance across a broad range of neuropsychological tests. Marginal improvement in a measure of auditory-verbal memory was observed while visuospatial memory declined. By contrast, renal transplantation, which led to near normalization of creatinine clearance, normalization of serum phosphorus and correction of anemia was associated with significant improvements in memory and learning with a trend towards improvement in psychomotor processing speed. When compared to 12 months of frequent nocturnal hemodialysis, renal transplantation was associated with a significant improvement in verbal learning and memory and in verbal fluency.

These results should be viewed in the context of whether the neurocognitive deficits seen in currently well-dialyzed patients with ESRD are reversible [21, 22]. In older studies, dialysis was shown to improve neurocognitive deficits seen in non-dialyzed patients [7]. However, recent evidence has raised concern that much of the residual cognitive dysfunction seen in contemporary well-dialyzed patients may be due to cerebrovascular disease and might not be reversible with further increases in solute clearance [21, 22]. Impairment of executive function is the most common neurocognitive deficit in contemporary dialysis patients [13]. A recent cross-sectional study found that compared with population norms, patients on hemodialysis have impaired executive function but not memory performance [14]. Defects in executive function have been linked with cerebrovascular disease and vascular dementia [23]. Imaging studies have confirmed that hemodialysis patients have more white matter lesions and cerebral atrophy than controls without CKD [24].

Using modern techniques of renal replacement therapy the relationship between dialytic clearance and cognition has been mixed. Cross-sectional studies of patients on maintenance hemodialysis have not found an association between variation in measures of uremia or dialytic clearance and cognition [13, 15]. However, the range of variation in clearance is relatively small. Cognitive performance has been reported to fluctuate temporally after hemodialysis with improvement in attention, concentration, verbal and visual memory and psychomotor speed 24 h after hemodialysis [25]. A significant decline in auditory memory and attention was reported after a weekend (67 h) without dialysis [8]. Switching from thrice weekly to short frequent daily dialysis did not improve cognitive performance in one small controlled study [16]. Alternatively, switching to frequent nocturnal hemodialysis was reported to improve psychomotor efficiency, attention and working memory in another small, uncontrolled study [17].

As previously reported, despite improvement in solute clearance, patients randomized to frequent daily or nocturnal hemodialysis in the FHN Trials did not improve global cognition compared to patients who remained on conventional thrice-weekly hemodialysis [19]. However, exploratory analyses in a subgroup of FHN patients who underwent a battery of neuropsychological tests similar to the present study, found that 12-months of frequent daily hemodialysis was associated with a significant improvement in memory and borderline improvement in verbal fluency compared to thrice-weekly hemodialysis [19]. By contrast, detailed neuropsychological testing in 21 patients randomized in the FHN Nocturnal Trial showed no improvement after 12-months of frequent nocturnal hemodialysis compared to conventional thrice-weekly hemodialysis. Surprisingly, in the Nocturnal Trial psychomotor processing speed at 12 months was found to be worse in patients randomized to frequent nocturnal hemodialysis compared to patients remaining on thrice-weekly hemodialysis.

The present study, included the 21 patients randomized in the FHN Nocturnal Trial and added an additional cohort of 56 patients, seven of which underwent frequent nocturnal hemodialysis. The results generally confirm and extend the observations from the FHN Trial, revealing that cognitive function, tested over multiple domains, did not improve one year after switching to frequent nocturnal hemodialysis compared to remaining on conventional thrice-weekly hemodialysis. However, in contrast to the FHN Trial the present study did not find that psychomotor processing speed was worse with frequent nocturnal hemodialysis compared to conventional thrice-weekly hemodialysis. This difference may be explained by the larger sample size and the fact that one patient randomized to frequent nocturnal hemodialysis had returned to conventional thrice weekly dialysis before the 4 month cognitive testing was performed and was analyzed “as treated” in the present study. These results also do not confirm a previous report that six months of frequent nocturnal hemodialysis significantly improved psychomotor efficiency, processing speed, attention and working memory [17]. Compared to the present study, the sample size in that study was smaller and their population was significantly younger with fewer comorbidities and did not include a control group. This might suggest that cognitive function in younger, healthier patients may respond better to frequent nocturnal hemodialysis but this would need to be examined further.

In contrast to dialysis, renal transplantation provides nearly complete correction of uremia as well as restoration of renal metabolic and endocrine functions. Consistent with the results of the present study, previous studies have reported that cognitive function in adult patients on dialysis can be improved by renal transplantation [9, 10, 26–28]. Improvements in verbal memory and psychomotor processing speed have been consistently observed while improvements in executive function and attention have been variable [9, 10, 26–28]. In contrast to the cognitive deficits seen in well-dialyzed adult patients on dialysis [11–14], cognitive function in renal transplant recipients has been reported to be indistinguishable from matched normal controls [26, 29]. Taken together, these results demonstrate that some of the cognitive deficits seen in well dialyzed adult patients, particularly in memory and psychomotor processing speed can be reversed by renal transplantation. Improvement in cognition with renal transplantation could be mediated by several factors including reversal of endocrine or metabolic dysfunction, correction of anemia [30, 31], better control of blood pressure, fluid and electrolytes. However, improvement in excretory function leading to better clearance of uremic toxins likely plays an important role in mediating the improvement in cognition following renal transplantation. The observed improvement of approximately 2 words in verbal learning and memory scores for transplant patients represents a clinically meaningful 19–35 % improvement over baseline and moves these scores into the normal range for age and education.

Taken together these results suggest that there are deficits in memory and verbal fluency in patients on thrice-weekly hemodialysis that can be improved by switching to frequent daily hemodialysis or receiving a renal transplant but not frequent nocturnal hemodialysis. Processing speed may also be improved by renal transplantation. In contrast, attention and executive function did not improve with frequent dialysis and has been inconsistently reported to improve following renal transplantation. While further work is needed, these results are consistent with the observation that impairment in executive function is associated with vascular disease and fixed structural lesions in the brain that are not readily reversible with increased solute clearance. The apparent lack of benefit of frequent nocturnal hemodialysis compared to frequent daily dialysis despite greater improvement in dialytic clearance was unexpected. This may be due to the small sample size of the studies. However, it is also possible that nocturnal dialysis may affect sleep patterns or otherwise adversely affect mental alertness that could counterbalance the positive effect of increased solute clearance on cognitive performance.

To our knowledge the present study is the largest study of the effect of frequent nocturnal hemodialysis on cognition. Notable additional strengths include the broad battery of neuropsychological tests employed, use of alternate test forms to minimize practice effects and the inclusion of both control groups for conventional thrice-weekly hemodialysis and renal transplantation. Nevertheless, the study was limited by patient dropout that truncated meaningful follow-up to an average of 12 months. In addition, the study population was relatively high functioning and the results may not generalize to general hemodialysis populations.

## Conclusions

For patients on conventional thrice-weekly hemodialysis receiving a functioning renal transplant was associated with improvement in auditory-verbal memory and psychomotor processing speed that was not observed after 12 months of frequent nocturnal hemodialysis. The lack of improvement in cognition with frequent nocturnal hemodialysis despite significant improvement in solute clearance was unexpected and requires further investigation.

### Availability of supporting data

The dataset for this study can be obtained by contacting the Bradley Dixon at bradley-dixon@uiowa.edu.

## Additional file

Additional file 1:
**Local institutional review boards (IRB) that approved study.** (DOCX 14 kb) Additional file 2: Table S1. List of baseline variables selected for the regression model on each cognitive test in Figure 2. (DOCX 18 kb)

## Abbreviations

AVLT: Auditory Verbal Learning Test
AVLT_LA_T1: Auditory Verbal Learning Test, word list A, first administration
AVLT_LA_T5: Auditory Verbal Learning Test, word list A, fifth administration
AVLT_LA_IR: Auditory Verbal Learning Test, word list A, immediate recall
AVLT_LA_DR: Auditory Verbal Learning Test, word list A, delayed recall
ANART: American National Adult Reading Test
BDI-II: Beck Depression Inventory
BVMT-R: Brief Visuospatial Memory Test –Revised
BVMT_T1: Brief Visuospatial Memory Test, first administration
BVMT_T3: Brief Visuospatial Memory Test, third administration
BVMT_DR: Brief Visuospatial Memory Test, delayed recall
CKD: Chronic kidney disease
COWAT: Controlled Oral Word Association Test
ESRD: End stage renal disease
FHN: Frequent Hemodialysis Network
Kt/V: measure of dialytic clearance
3MS: Modified Mini-Mental Status Exam
StdKt/V: standardized dialytic clearance
