# Supplementary material for: Cognitive changes associated with switching to frequent nocturnal hemodialysis or renal transplantation
Source: BMC Nephrol. 2016 Jan 22;17:12. doi: 10.1186/s12882-016-0223-9 (PMC4722762; doi:10.1186/s12882-016-0223-9)
Supplement: Additional file 1: — Local institutional review boards (IRB) that approved study. (DOCX 14 kb) [file 12882_2016_223_MOESM1_ESM.docx]

Additional File 1

Local institutional review boards (IRB) that approved study:

University of Iowa IRB-01

Cleveland Clinic Institutional Review Board

Committee on Clinical Investigations, Beth Israel Deaconess Medical Center

University of Virginia IRB – Health Sciences Research and Centra Health IRB

Office of Research Ethics, Western University, Canada

University of Toronto Research Ethics Board

Wake Forest Health Sciences IRB
