# Supplementary material for: Cognitive changes associated with switching to frequent nocturnal hemodialysis or renal transplantation
Source: BMC Nephrol. 2016 Jan 22;17:12. doi: 10.1186/s12882-016-0223-9 (PMC4722762; doi:10.1186/s12882-016-0223-9)
Supplement: Additional file 2: Table S1. — List of baseline variables selected for the regression model on each cognitive test in Figure 2. (DOCX 18 kb) [file 12882_2016_223_MOESM2_ESM.docx]

**Additional File 2.**

**Supplemental Table S1: List of baseline variables selected for the regression model on each cognitive test in Figure 2.**

|  |  |  |  |  |  |
| --- | --- | --- | --- | --- | --- |
| **Test** | **Confounders** |  |  |  |  |
| AVLT_LA_T1 | Age, Gender |  |  |  |  |
| AVLT_LA_T2 | Pre Systolic Blood Pressure, Gender |  |  |  |  |
| AVLT_LA_T3 | Vascular Disease Indicator, Age, Gender |  |  |  |  |
| AVLT_LA_T4 | Age, Gender, Time on dialysis before baseline |  |  |  |  |
| AVLT_LA_T5 | Pre Systolic Blood Pressure, Gender, Diabetes Indicator, Age, Time on dialysis before baseline |  |  |  |  |
| AVLT_LB_T1 | Diabetes, Gender |  |  |  |  |
| AVLT_LA_IR | Age, Time on dialysis before baseline |  |  |  |  |
| AVLT_LA_DR | Age, Time on dialysis before baseline |  |  |  |  |
| BMVT_T1 | Age |  |  |  |  |
| BMVT_T2 | Erythropoietin Usage Indicator, Age, Gender |  |  |  |  |
| BMVT_T3 | Pre Systolic Blood Pressure, Erythropoietin Usage Indicator, Age, Race category (White vs Other) |  |  |  |  |
| BMVT_DR | Pre Systolic Blood Pressure, Erythropoietin Usage Indicator, Potassium, Age, Gender, Race Category |  |  |  |  |
| Digit Symbol | Diabetes, Anticonvulsant Indicator, H1Receptors Indicator, Serum Albumin, Age, Gender |  |  |  |  |
| Trails A | Age, Race category |  |  |  |  |
| Trails B | Vascular Disease Indicator, Age, Gender, H1 Receptors Indicator |  |  |  |  |
| Letter Number Sequence | Serum Albumin, Age, Gender |  |  |  |  |
| Verbal Fluency | Urea Reduction Ratio, Education |  |  |  |  |
| Chooser | Anticonvulsant Indicator, Age |  |  |  |  |
| Buttons | Vascular Disease Indicator, Stroke Indicator, Serum Albumin, Age, Race Category |  |  |  |  |
| N-Back | Serum Albumin |  |  |  |  |

See Table 4 for abbreviations.
